# Structural Constraints Identified with Covariation Analysis in Ribosomal RNA

**DOI:** 10.1371/journal.pone.0039383

**Published:** 2012-06-19

**Authors:** Lei Shang, Weijia Xu, Stuart Ozer, Robin R. Gutell

**Affiliations:** 1 Institute for Cellular and Molecular Biology, Center for Computational Biology and Bioinformatics, The University of Texas at Austin, Austin, Texas, United States of America; 2 Texas Advanced Computing Center, The University of Texas at Austin, Austin, Texas, United States of America; 3 Microsoft Corporation, Redmond, Washington, United States of America; MRC National Institute for Medical Research, United Kingdom

## Abstract

Covariation analysis is used to identify those positions with similar patterns of sequence variation in an alignment of RNA sequences. These constraints on the evolution of two positions are usually associated with a base pair in a helix. While mutual information (MI) has been used to accurately predict an RNA secondary structure and a few of its tertiary interactions, early studies revealed that phylogenetic event counting methods are more sensitive and provide extra confidence in the prediction of base pairs. We developed a novel and powerful phylogenetic events counting method (PEC) for quantifying positional covariation with the Gutell lab’s new RNA Comparative Analysis Database (rCAD). The PEC and MI-based methods each identify unique base pairs, and jointly identify many other base pairs. In total, both methods in combination with an N-best and helix-extension strategy identify the maximal number of base pairs. While covariation methods have effectively and accurately predicted RNAs secondary structure, only a few tertiary structure base pairs have been identified. Analysis presented herein and at the Gutell lab’s Comparative RNA Web (CRW) Site reveal that the majority of these latter base pairs do not covary with one another. However, covariation analysis does reveal a weaker although significant covariation between sets of nucleotides that are in proximity in the three-dimensional RNA structure. This reveals that covariation analysis identifies other types of structural constraints beyond the two nucleotides that form a base pair.

## Introduction

Covariation analysis, one form of comparative analysis, identifies the positions in the RNA molecule that have similar patterns of variation, or covariation, for all or a subset of the sequences within the same RNA family. It was initially utilized to predict the cloverleaf secondary structure for tRNA [1] which was subsequently verified with high-resolution crystallography [2], [3]. A few other examples of RNA molecules that were predicted with comparative analysis and verified with high-resolution crystallography are the 5S, 16S, and 23S rRNA [4], [5], [6], group I introns [7], [8], [9], RNase P [10], [11], [12], tmRNA [13], [14], U RNA [15], [16], and SRP RNA [17], [18], [19]. These examples provide additional support that comparative analysis can identify the secondary structure for some RNAs with extremely high accuracy.

While the earliest covariation analysis methods searched for G:C, A:U, and G:U base pairs that occur within a regular secondary structure helix [1], [20], [21], [22], newer more mathematically and computational rigorous methods primarily searched for columns in an alignment of sequences for similar patterns of variation, based on their nucleotide frequencies, regardless of the type of base pair and the location of each putative base pair in relation to the other base pairs [23], [24], [25], [26]. These latter studies had a simple and profound result. The vast majority of all base pair types were canonical - G:C, A:U, and G:U, and these base pairs were consecutive and antiparallel to form a regular helix. Thus this structure agnostic method for the identification of positional covariation had independently identified two of the most fundamental principles of RNA structure – the two base pair types initially determined by Chargaff [27], [28], and Watson and Crick [29], and the arrangement of these base pair types into regular nucleic acid helical structures [29]. However, this search for positions in an alignment with similar patterns of variation have also identified numerous non-canonical base pair exchanges [30], [31], pseudo-knots [31], [32], base triples [33], [34], [35], and sets of positions with a weak network of covariations [26], [33]. Thus, while the vast majority of the nucleotide positions with a very strong covariation form a canonical base pair within a standard helix, a small number of significant covariations are not part of a regular helix and do not exchange solely between canonical base pair types.

The traditional methods to identify positional covariation utilize the nucleotide frequencies for each of the base pair types. While this approach has been very successful, as discussed earlier, the phylogenetic relationships between the sequences can enhance the sensitivity for the determination of the number of mutual changes that have occurred during the evolution of the RNA. Our confidence in one of the first putative helices that forms a pseudo knot was significantly bolstered when we determined that several of the same base pair types (e.g. A:U, G:C) had evolved multiple times in the evolution of the 570/866 base pair in 16S rRNA [32], thus increasing the likelihood that these two positions with similar patterns of variation did not occur by chance. Accordingly, our analysis of the sequences in hairpin loops with four nucleotides (commonly called – tetraloops) revealed hairpin loops in the 16S rRNA that frequently changed between GNRA, UUCG, and CUUG [36] during the evolution of the rRNA. Thus the evolutionary history of the sequences and the positions within the sequences is another dimension of information that enhances the resolution and alternative interpretations of the covariation analysis. For these two studies published in 1986 and 1990, the numbers of phylogenetic events - coordinated changes during the evolution of the RNA, were counted from a visual inspection of the data. However, new computational methods are essential to automatically identify covariations based on phylogenetic relationships. Several papers have been presented that identify covariations based on modeling phylogenetic relationships [37], [38], [39].

The Gutell lab developed a novel and sophisticated multidimensional relational database system for the comparative analysis of RNA. This system was named rCAD – **R**NA **C**omparative **A**nalysis **D**atabase [40]. It integrates and cross-indexes several dimensions of information for storage, retrieval, and analysis. While this infrastructure has multiple applications for the analysis of the structure, function, and evolution of RNA, for the objectives of this study, we have utilized rCAD to determine the changes at each position in the RNA molecule during its evolution. This Phylogenetic Event Counting (PEC) method traversals the phylogenetic tree hierarchy and measures the significance of covariations between two positions. A Joint N-Best method and a Helix-extension procedure are utilized to enhance the identification and accuracy of identification of the structural constraints present in the sequence alignment. A comparison between our PEC based method and other covariation methods reveals that while PEC is overall superior in the identification of base pairs, MI based methods identify unique base pairs, and they jointly identify many other base pairs. Both types of methods, when applied simultaneously identifies even more base pairs than either method by themselves. And last, these covariation methods also identify other types of constraints in an RNA structure.

## Results

### 1. Conceptual Overview of the Methods Used in Analysis

#### 1.1. The phylogenetic event counting method

**Figure 1:**
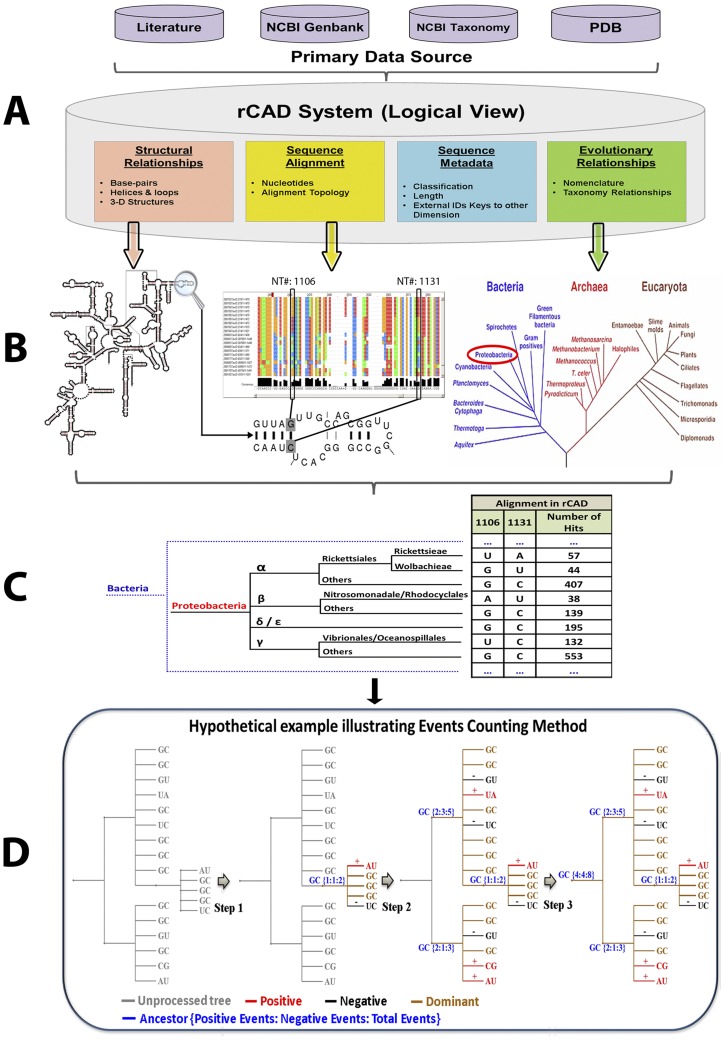
The highlight and underlying concepts of the PEC based covariation analysis in rCAD. A: data source; B: multi-dimensional data; C: mapping the substitutions; D: counting the positive and negative events.

The overall analysis workflow of the Phylogenetic Event Counting method (PEC) is shown in Figure 1. The four primary forms of data are: (1) metadata, including functional information about sequences and structures; (2) sequences and sequence alignment; (3) higher-order structure and (4) evolutionary/phylogenetic relationships between the sequences and structures are stored and analyzed in rCAD (Figure 1A & 1B). For each pair of positions under consideration, the nucleotides are mapped onto the phylogenetic tree (Figure 1C). A tree-traversal from leaf nodes to root counts all positive events (both position change from child node to its direct parent node) and negative events (only one position changes) (Figure 1D). The nucleotides of ancestor nodes are determined by using maximum parsimony strategy. However, to avoid bias caused by repeat sampling in certain branches, each type of pair of child nucleotides is counted only once. For example, ancestor node is {U:A}, child nodes contain U:A which occurs 10 times, A:U which occurs 2 times, A:C which occurs 1 time. The A:U pair will be counted only once as positive events regardless of its actual occurrence. Thus we minimize the observed events in our analysis to assure high confidence. The pseudo code of PEC algorithm is described in Figure S1. The covariation between two positions is determined by calculating the Covariation Percentage of Events (CPE), the ratio of positive events to the total number of events (positive and negative) (Details in Method section).

#### 1.2. Base pair identification process

**Figure 2:**
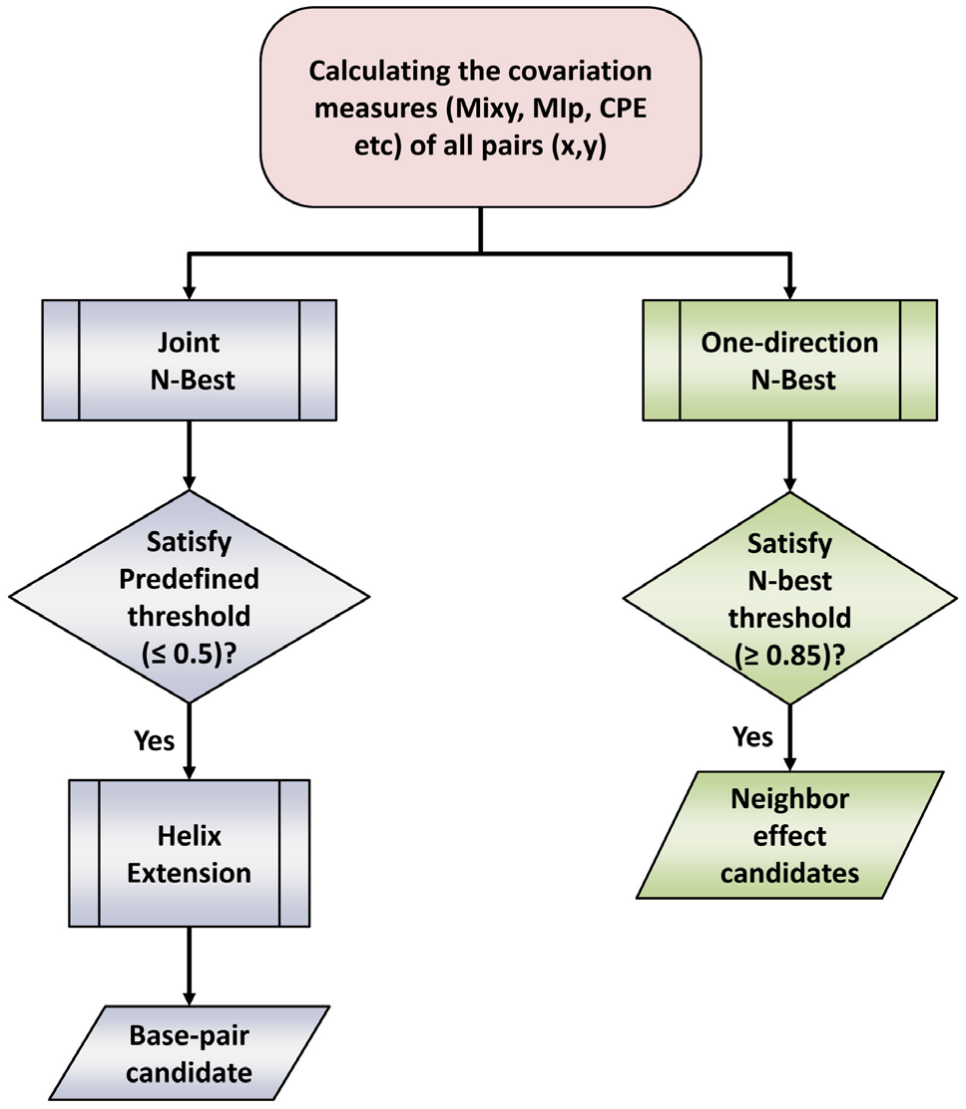
The flowchart of analysis in the identification of base-pairs and neighbor effects.

The analysis procedure that reveals structural constraints of RNA molecules is presented in Figure 2. Base pairs with covariations are identified with a Joint N-Best strategy which measures the significance of covariation score between the two positions, followed by a helix extension procedure to further improve the sensitivity (Process colored blue in Figure 2).

The N-Best strategy was initially used with mutual information (MIxy) on a set of tRNA sequences [26]. MIxy values increase for similar extents of covariation as the entropy value decreases (ie. increases in variation, the MIxy values should be standardized for the different entropy values). A simple method to approximate this is to rank the positions with the highest mutual information scores, or covariation for each individual position. For the majority of base pairs in the comparative structures for the tRNAs [26], the positions that form a base pair with the cardinal position number usually has a MIxy value significantly higher than the MIxy values for the other ranked positions.

This standardizes the covariation scores by first ranking the positions with their MIxy values, followed by the calculation of a ratio value of the second highest covariation score to the highest score. Our confidence in the prediction of a base pair is proportional to the difference between the two positions with the largest values. The likelihood that position X is base paired to position Y is further enhanced when the position with the highest score for X is Y, and when the position with the highest score for Y is X. This Joint N-Best with PEC method (PEC/JN-Best) improves the sensitivity and accuracy of the base-pair identification (see the Method section). Pairs of positions satisfying a predefined N-Best threshold (≤0.5) are considered as base pair candidates with significant covariations.

The amount of covariation is directly proportional to the amount of variation in a sequence alignment. Figure S2 reveals the relationship between the overall variation in the bacterial 16S rRNA alignment and the amount of variation in three categories in the secondary structure: 1) both positions involved in base pairs, 2) one of the two base paired positions, and 3) the unpaired positions.

This variation/covariation analysis reveals that more conserved positions are less likely to be identified as a base pair with covariation methods than positions that have more variation (ie – no variation, no covariation). Since our objective is to identify all of the secondary structure base pairs, the base pair candidates identified with Joint N-Best strategy are used as the nucleation pairs in the helix extension process. The helix extension increases the length of a putative helix with G:C, A:U, and G:U base pairs that are 1) adjacent and antiparallel with the nucleation pair and 2) occur in at least 85% of the sequences. A less quantitative version of this helix extension was first applied in the original Noller-Woese 16S and 23S rRNA secondary structure models [20], [41]. A helix with the maximal number of G:C, A:U, and G:U base pairs was formed when at least one base pair had a covariation. As more 16S and 23S rRNA sequences were determined, some of the extended base pairs from the nucleation base pair were removed when the two positions did not have similar patterns of variation while the majority of the extended base pairs did have similar patterns of variation in alignments that contained more sequences [6], [25]. Our confidence in a predicted base pair is directly proportional to the amount of covariation. Thus, we have less confidence in those base pairs that have minimal or no covariation.

The high-resolution crystal structure of the *T. thermophilus* 30S ribosomal subunit (PDBID: 1J5E) which contains the 16S rRNA and E. Coli 50S ribosomal subunit (PDBID: 2AW4)are the reference structures for this study. All identified covariant pairs are categorized as either true positives (annotated in the reference crystal structure) or false positives (not annotated in the reference crystal structure).

#### 1.3. Neighbor effects identification process

Previous analysis has revealed that when two positions in a sequence alignment have very similar patterns of variation, as gauged with a high covariation score, those positions usually form a base pair in the RNA higher-order structure. However as the extent of positional covariation decreases, our observations here and in our previous analysis [26], [33] reveals that some pairs with lower covariation scores form base pairs, and others do not. While the full significance of these observations have not been determined, we have observed that the positions in these clusters of significant but lower covariation scores are usually very close with one another in the three-dimensional structure with the traditional, covariation methods, hereafter named neighbor effects [33], [42].

Figure S3 shows that the highest covariation score for the majority of all positions that are base paired is significantly higher than the position with the second best score (example of nucleotides 3 in tRNA are presented in S3a left side, while the overall picture are shown in S3b). However, the highest covariation value for some base pairs is lower while the set of next highest positions are closer to the highest (see Figure S3a right side and Figure S4).

We identify a set of “neighbor effects” using a standard one-directional N-best method with some covariation and structural constraints (Process colored green in Figure 2, details in the Method section).

### 2. Application of the Methods on Datasets

#### 2.1. The datasets used and the strategy of reducing the number of pairwise comparisons

Three data sets are used in this analysis: a bacterial 16S rRNA sequence alignment containing 4142 sequences with 3236 Columns (Dataset S1); a bacteria 5S rRNA alignment containing 2088 sequences with 333 columns (Dataset S2); and a bacteria 23S rRNA alignment containing 2339 sequences with 7330 columns (Dataset S3). The sequences in this analysis include organisms from most of the major branches of the bacterial phylogenetic tree (details in Table S1).

The significance of this covariation analysis is dependent on the accuracy of the alignment of sequences. We have utilized alignments from the Comparative RNA Web (CRW) Site [6]. These alignments are the culmination of more than twenty years of refinement. Starting with sequences that have sufficient sequence identity, covariation analysis was used to predict the early secondary structure models that were subsequently used to refine the alignment in parallel with the addition of more sequences. Additional covariation analysis with more sophisticated algorithms were used to refine the secondary structure in the regions of the rRNA that are present in all of the sequences, regions present in just the major phylogenetic domains (ie. Archaea, Bacteria, and Eucarya), present in sub-branches within these three domains, etc. This process resulted in secondary structure models that are very accurate. A total of 97–98% of the base pairs predicted with comparative analysis are in the high-resolution crystal structure [43]. This high accuracy substantiates the accuracy of the sequence alignments and the subsequent covariation analysis. A more detailed description of the alignment of RNA sequences have been published [6], [25], [30].

The *Escherichia coli* [44] is the typical reference sequence for 5S, 16S, and 23S rRNA comparative structure models. The high-resolution three-dimensional structure for *Thermus thermophiles* 30S ribosomal subunit [5] is utilized in the analysis of the 16S rRNA while the high-resolution structure for *Escherichia coli* 50S ribosomal subunit [45] is used in the analysis of the 5S and 23S rRNA. The sequences in these crystal structures are used as the reference sequences. To expedite the phylogenetic event counting method, only those pairwise positions that have the likelihood of having a significant covariation were analyzed. The process of selecting those sets of positions is illustrated for 16S rRNA. This sequence has 1521 nucleotides, while the alignment contains 3,236 columns. Every column in the alignment is analyzed with every other column. Thus the total number of pairwise comparisons is 5,234,230. The time complexity of PEC algorithm on this dataset scales to O(4.4×10^10^). The PEC algorithm requires a significant amount of time to transverse the entire phylogenetic tree and count the number of changes during the evolution of the RNA. Since the positions with similar conservation scores have the higher probability to have good covariation score (details in Figure S5), the number of pairwise comparison calculations is reduced significantly by analyzing only those sets of positions with similar conservation scores., Therefore a coarse filter is applied to reduce the number of pairwise comparison to 14,276 ([46], details in Method section, a complete list in Table S2).

#### 2.2. Performance comparison of different covariation methods in the identification of base pairs

The performance of our PEC method in the identification of real base pairs -is compared with other published methods using the bacterial 5S, 16S, and 23S rRNA alignment data sets. The percentage of predicted base pairs that are present in the crystal structures are measured as a function of rank order using a variety of methods: PEC, MIxy [24], [26], MIp [47], OMES [48], ELSC [49], and McBASC [50]. In addition to the methods that are used here to evaluate the performance of our PEC method, we also tried to evaluate several other programs including PSICov [51], Direct information (DI) [52], RNAalifold [53], RNAfold [54], [55], Pfold [56], [57]and Evofold [58]. However, these programs are either not suitable for the prediction of higher-order structure of RNA with covariation analysis or they are unable to operate on the large alignments used in our study.

**Figure 3:**
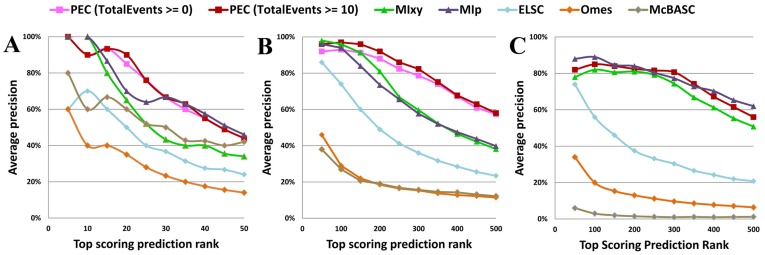
The precision of top N ranked prediction plot with different covariation methods in the identification of base pairs using different data sets. A: 5S rRNA data set; B: 16S rRNA data set; C: 23S rRNA data set.

The precision of top N ranked prediction plot, utilized in several similar covariation analysis studies [47], [51], [59], [60] to gauge the precision of several covariation methods, is shown in Figure 3. These plots reveal the fraction of pairs with ranked N or higher in each data set that are the contacting base pairs in the crystal structures. For the 16S rRNA alignment (Figure 3B), the PEC method performs better than Mixy and MIp, and significantly better than ELSC, OMES, and McBASC. For the 5S and 23S rRNA alignments, PEC and the MIp have higher values that are similar with one another, while the values for the ELSC, OMES, and McBASC methods are considerably lower (Figure 3A and 3C). The total event (positive events plus negative events) measures the total number of changes on a pair of positions throughout their evolution. Adding the total event threshold (e.g. > = 10) reduces background noise and improves the accuracy of PEC method. As shown in Figure 3A and 3B, PEC with total events threshold achieves higher accuracy than PEC without total events threshold. However, that performance of PEC with or without total events threshold is exactly the same on the 23S rRNA data set (Figure 3C). Overall while the PEC method is superior, MIp is the second best method in identifying base pairs.

#### 2.3. Application of joint N-best

The precision of top N ranked curve plot in Figure 3 reveals that the PEC, MIp, and MIxy methods are the top 3 solutions for our data sets. Mutual information (MIxy) measures the dependence between two positions in the RNA sequence alignment. It was first introduced for the identification of base pairs in RNA [24], [26]. Lindgreen et al. evaluated 10 different mutual information based methods for the identification of covariations in RNA alignments [61]. While they demonstrated that the standard implementation of MIxy is a good measure for the prediction of base pairs in the secondary structure, several variations of the simple implementation improved the prediction of the base pairs. Additional improvements in the implementation of Mixy [47] utilized a method (MIp) that estimates the level of background mutual information for each pair of positions. After removing the background and introducing a Z-score (MIp/Z-score), Dunn et al. [47] have determined that their MIp/Z-score method identified substantially more co-varying positions than other existing mutual information based methods.

In our analysis, we use Joint N-best algorithm to determine the significance of the covariation scores calculated in different methods (details in Methods section). The Joint N-best algorithm is used with PEC, MIxy, and MIp methods (PEC/JN-Best, MIp/JN-Best, MIxy/JN-Best). The recommended (default) cutoff value of N-best score is 0.5. We also make a conversion from MIp to Z-score (MIp/Z-score) with the recommended Z-score cutoff as comparison [47].

**Figure 4:**
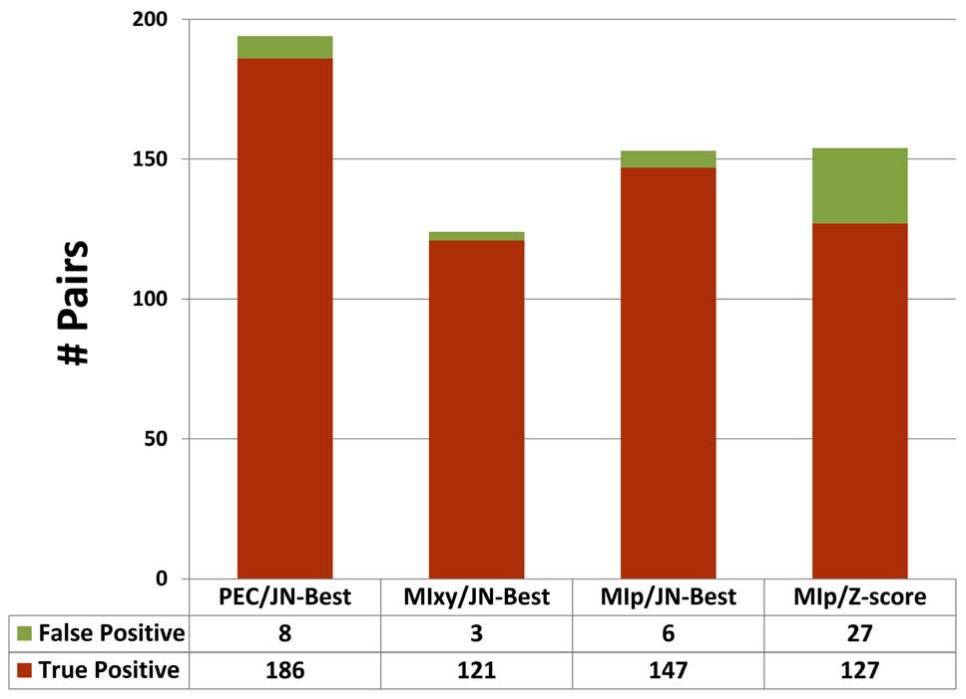
The number of true positives and false positives identified in different methods.

The PEC/JN-Best, MIxy/JN-Best and MIp/JN-Best methods are used on the 5S, 16S and 23S rRNA data sets. For the 16S rRNA (Figure 4), the PEC/JN-Best method identifies 186 real base-pairs (true positives) with only 8 false positives (95.9% accuracy), while the MIxy/JN-Best identifies 121 true positives with 3 false positives (97.6% accuracy). The MIp/Zscore method identifies 127 true positives however the number of false positives –27 decreases the accuracy (82.5%). Utilizing the Joint N-Best method with the MIp (MIp/JN-Best) increases the number of true positives to 147 and decreases the number of false positives to 6. This MIp/JN-Best method identifies all but one pair found by MIxy/JN-Best. Thus, with the default N-best cutoff (0.5), the PEC/JN-Best method has higher sensitivity and accuracy than MIxy/JN-Best and MIp/JN-Best in detecting covariant base pairs.

Since MIp/JN-Best method identifies all of the pairs found by the MIxy/JN-Best method except for the pair 150∶159 (*Thermus thermophiles* numbering), we combine the non-redundant pairs identified in both methods. These pairs are referred as identified by Mutual Information Based Measure with Joint N-Best (MI/JN-Best).

**Figure 5:**
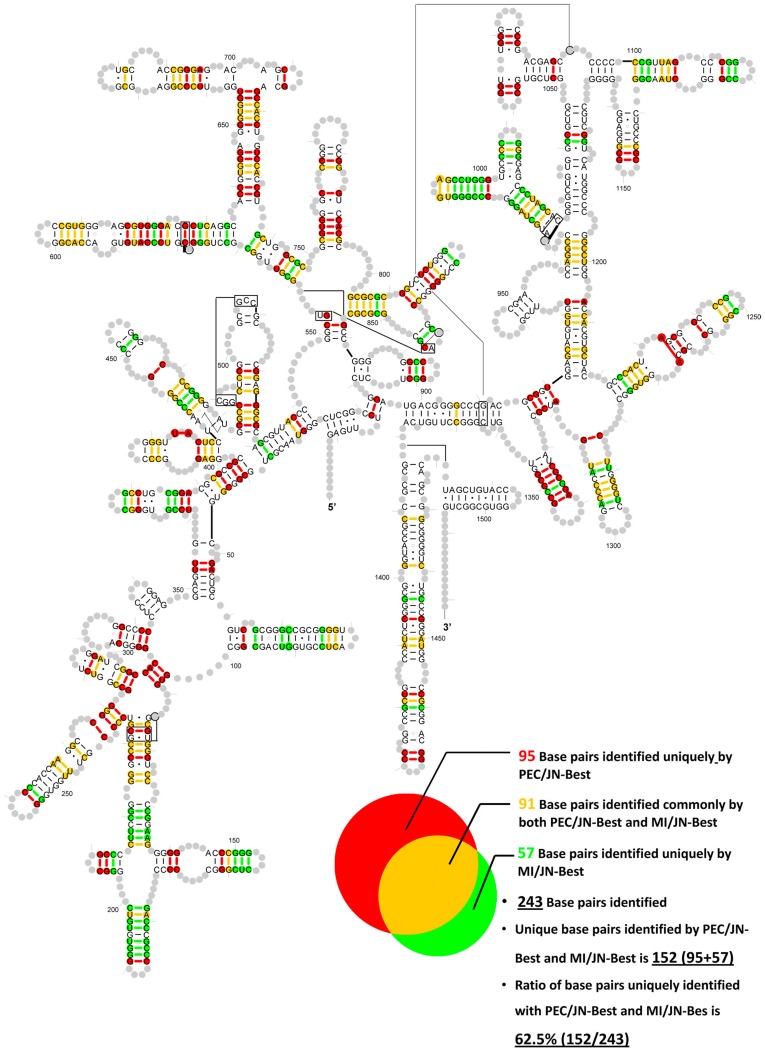
The base pairs (true positives) identified by PEC/JN-Best and MI/JN-Best are plotted onto the *T. thermophiles* 16S rRNA secondary structure diagram. Red: base pairs only identified by PEC/JN-Best; Green: base pairs only identified by MI/JN-Best; Yellow: base pairs identified by both methods.

The real base-pairs (true positives) identified by PEC/JN-Best and MI/JN-Best methods are plotted onto the 16S rRNA secondary structure diagram (Figure 5). The distribution of base pairs only identified by PEC/JN-Best, only by MI/JN-Best, and by both methods are: 95 (red), 57 (green) and 91 (yellow). The total number of base pairs identified is 243. The ratio of the number of base pairs that are uniquely identified with PEC/JN-Best and MI/JN-Bes is 62.5%.

A general comparison of these methods for 5S, 16S, and 23S rRNA (Table S3) reveals: 1) while both PEC/JN-Best and MI/JN-Best identifies base pairs not identified with the other method, both methods also identified many of the same base pairs, 2) MIp/JN-Best was superior to the MIp/Z-score for the 16S rRNA with the default settings, and 3) MIp/JN-Best identifies a larger percentage of the base pairs found with by MIxy/JN-Best.

#### 2.4. Identification of highly conserved base pair with helix-extension strategy

**Figure 6:**
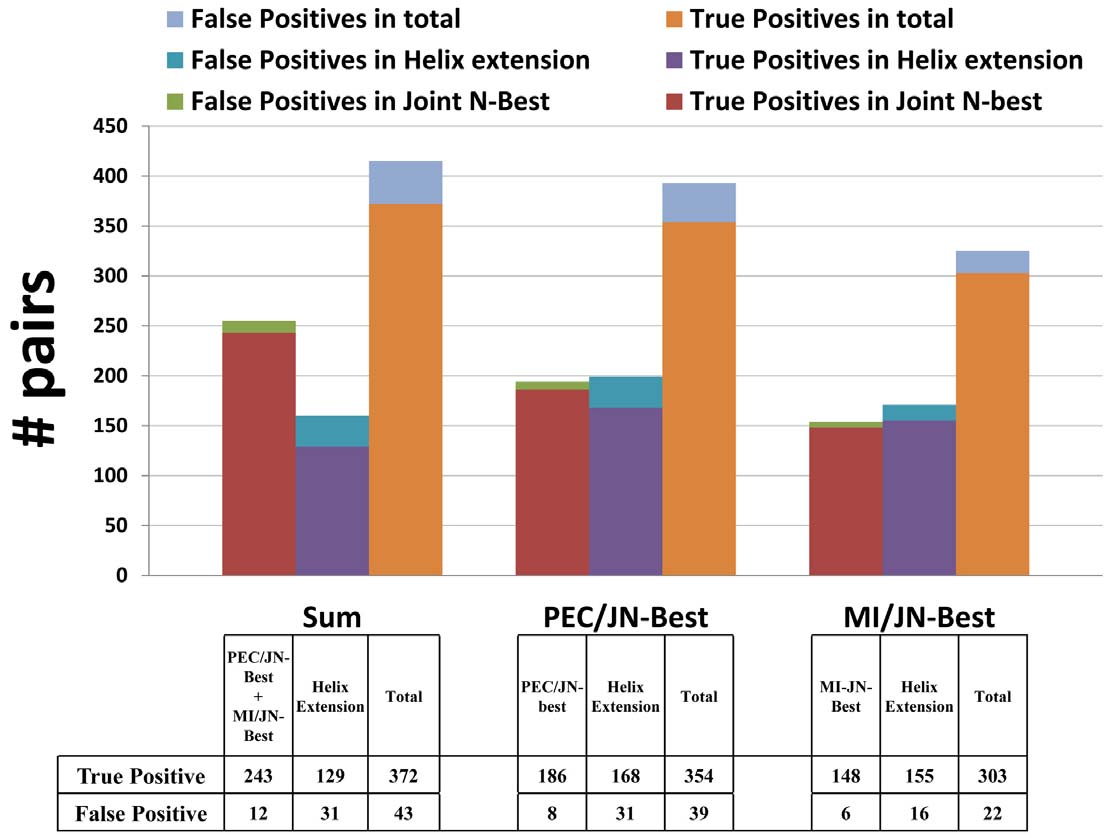
For each method, the number of true positives and false positives identified in the Joint N-Best calculation (nucleation pairs), following helix extension procedure (extended pairs), and sum of them are shown as a stacked histogram.

**Figure 7:**
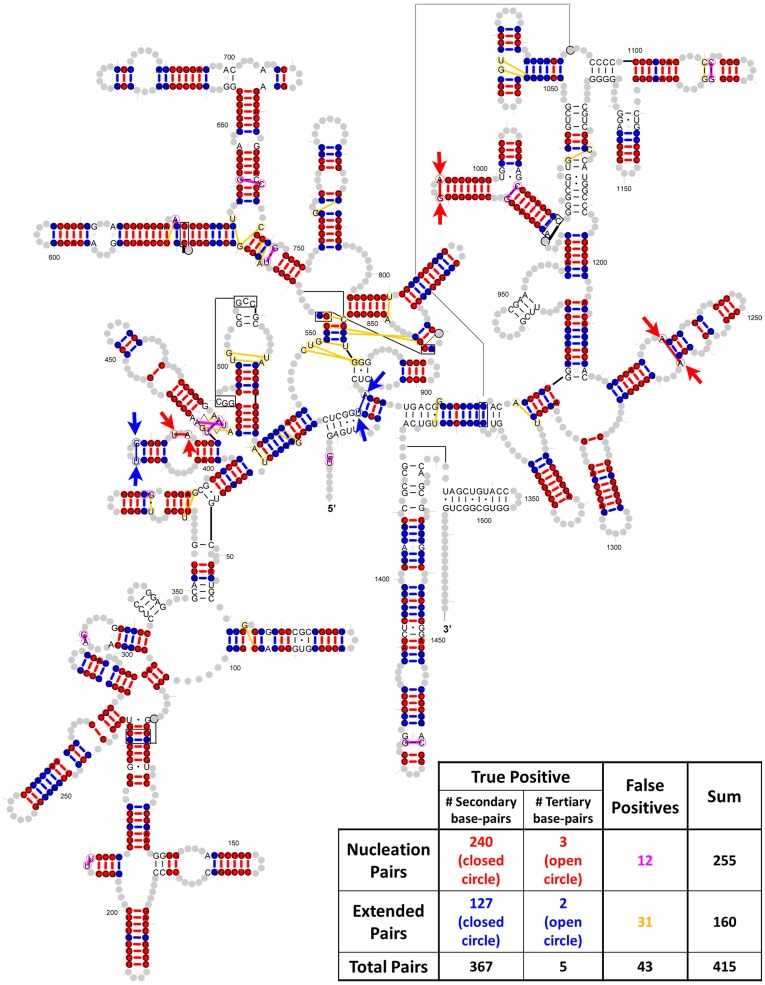
Base pairs in the Bacterial 16S rRNA structure model that are identified with the helix extension method. Red: true positive base-pairs identified as the sum of PEC/JN-Best and MIxy/JN-Best methods, which are used as nucleation points in the helix extension Magenta: false positives in the nucleation pairs; Blue: true positive base-pairs identified with the helix-extension method; Yellow: false-positive pairs identified with the helix-extension method. Secondary base-pairs are represented by closed circles while tertiary base-pairs are represented by open circle and highlighted with arrows.

The sum of non-redundant predicted base pairs by PEC/JN-Best and MI/JN-Best methods in 5S, 16S, and 23S rRNA datasets are used as nucleation pairs in the helix-extension procedure. Extended pairs are composed of the nucleotides that are adjacent and antiparallel to the nucleation pair. All extended pairs have more than 85% WC/Wobble base-pair nucleotides in the alignment. Additional base pairs that satisfy this helix extension threshold continue to be added to this extending helix until they fail the extending threshold. A complete list of pairs involved in helix extension is shown in Table S4. For 16S rRNA data set (Figure 6 left), the total number of base pairs added with the helix extension is 160; 129 of these are present in the crystal structure, while the 31 false positives primarily occur at the end of helices. The nucleation and extended pairs are mapped onto the secondary structure diagram of *T. thermophilus* 16S rRNA, as shown in Figure 7. The number of nucleation pairs with PEC/JN-Best and MI/JN-Best, the extended pairs in the helix extensions - and the secondary structure diagrams are shown in Figure 6 (middle and right), and Figure S6 respectively. This result demonstrates that with a collection of high-quality nucleation pairs, the helix extension strategy is accurate and sensitive in the identification of highly conserved base pairs. The successful application of this Helix-extension method onto the 5S and 23S rRNA data sets further substantiates this conclusion (A complete list in Table S4).

#### 2.5. The purity of the secondary and tertiary structure base pairs in the crystal structure compared with the conservation scores

Most of the base pairs identified are part of the secondary structure. Of these, 240 are identified with the Joint N-best analysis and 127 are found with the helix extension procedure for the 16S rRNA data set (represented as closed circle in Figure 7). Only a few tertiary structure base pairs are identified: 3 in Joint N-best and 2 in helix extension procedure (represented as open circle and get highlighted by arrows in Figure 7).

**Figure 8:**
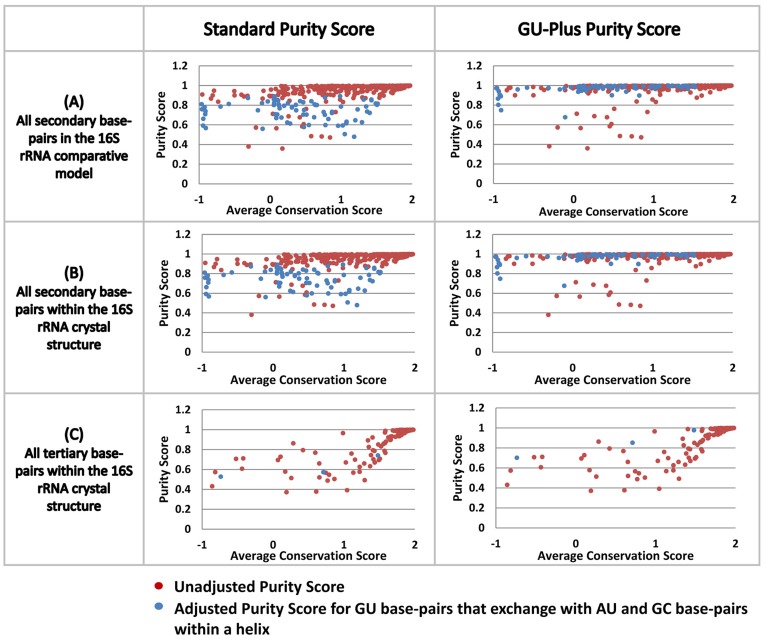
The distribution of purity score and average conservation (or informational entropy) for the two nucleotides that form a base pair in the 16S rRNA comparative structure model (A), secondary structure base pairs in crystal structure (B), and tertiary interactions in crystal structure (C).

A quantitative and graphical analysis illustrates the general observation noted in the previous paragraph – secondary structure base pairs usually have strong covariation between the two nucleotides that form that interaction while the majority of the tertiary structure base pairs have weak or no covariation between the two nucleotides that form that interaction. The purity score – a measure of the precision of covariation (details in Method section and Figure S7), is plotted against the conservation score (or informational entropy, see Method section) for the two positions that form a base pair (Figure 8). This analysis was performed for the 16S rRNA comparative secondary structure and the high resolution crystal structure for *Thermus thermophilus* 16S rRNA. For both of these molecules, two plots were created, the first for the unaltered purity score and the second for purity scores adjusted for G:U base pairs (see Methods section; Figure 8). Base pairs in the bacterial 16S rRNA dataset range from highly conserved to highly variable in the comparative and crystal structures. The overall results from these plots are as expected: (1) The majority of the secondary structure base pairs are at or very close to a purity score of 1; (2) Many of the base pairs with a lower absolute purity score increase their GU-plus score to or near 1, indicating that many of the base pairs associated with these lower purity scores involve a G:U base pair; (3) The majority of tertiary structure base pairs do not have the highest purity scores, indicating that many of positions that form tertiary base pairs have no covariation, or some covariation with many exceptions, consistent with our previous observation [43] [http://www.rna.ccbb.utexas.edu/SAE/2A/xtal_Info/16S/Index].

#### 2.6. The identification of “neighbor effects”

As shown in earlier sections of this manuscript and previous studies [37], [38], phylogenetic event-based covariation methods have the potential to identify covariations that are not observed with the traditional methods.

The covariation values for the highest and second highest positions for the base pairs identified in our PEC/JN-Best method are significantly different (threshold value of 0.5, see “The Joint N-Best strategy” in the Methods section). These base pairs are analogous to the tRNA base pair 3∶70 in Figure S3a left side. However the difference between the highest and the set of next highest positions in our Bacterial 16S rRNA dataset are smaller for numerous positions, analogous to Figure S3a right side and Figure S4. As first defined in [26], positions with these characteristic covariation values are referred to as neighbor effects, and are usually physically close to one another. Neighbor effects are defined herein as those positions with the N-best scores exceeding a predefined threshold of ≥0.85 (see Methods section) and are in close proximity. For this manuscript, the physical distance is minimal for those positions that are defined to be a neighbor effect. This criterion is satisfied for those positions with at least 10 phylogenetic events (Figure S8).

**Figure 9:**
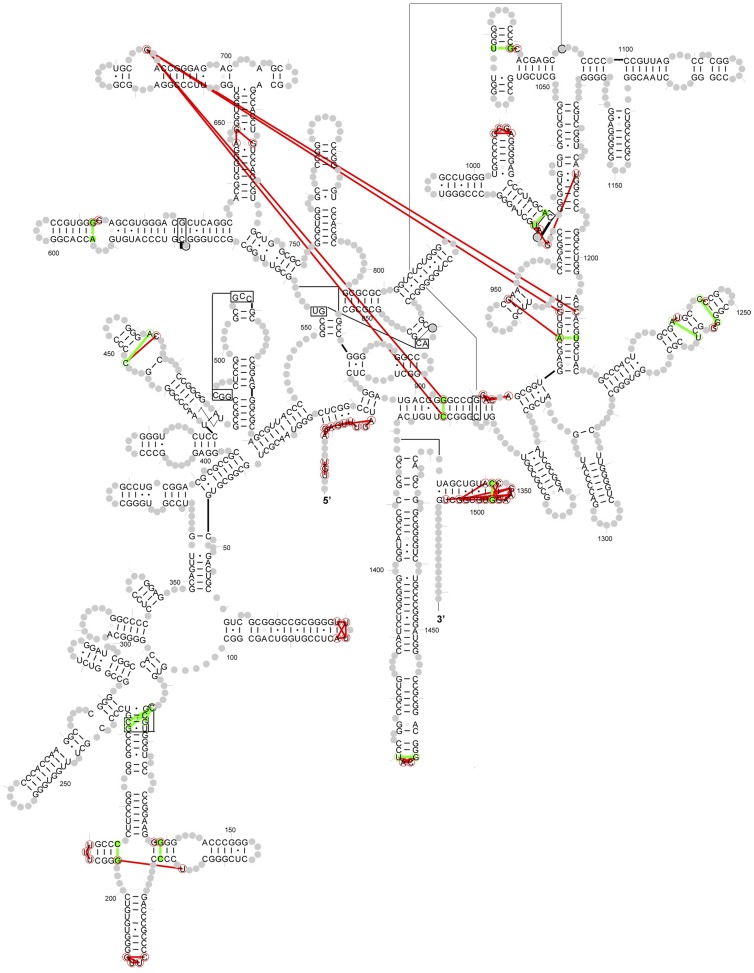
The secondary structural diagram of *T. thermophilus* 16S rRNA reveals all identified neighbor effects. Red lines connecting nucleotides indicate non base-pairing interactions. Green lines represent the base-pairs or base-triples identified as neighbor effects.

With this criteria, 89 neighbor-effect pairs are identified and plotted onto the secondary structure diagram of *T. thermophilus* 16S rRNA (Figure 9, a complete list in Table S5). Among the 89 neighbor effect pairs, 15 have hydrogen bonding between the nucleotides in the 16S *T. Thermophilus* rRNA crystal structure (8 secondary base-pairs, 4 tertiary base-pairs and 3 base-triples). These are colored green in Figure 8. The remaining 74 pairs do not form hydrogen bonds between the bases. These are colored red. Of the 89 neighbor effects pairs, only four (686∶905, 686∶930, 686∶1209 and 686∶1371, *T. thermophiles* numbering) are separated by more than 30 Å. The average distance between these neighbor effects is 8.82±5.91 Å. Most of these neighbor effects involve nucleotides that are either consecutive on the sequence, each nucleotide of the pair are on opposite sides of a helix, adjacent to two nucleotides that form a base pair, or involve a nucleotide in a loop and a nucleotide in a helix that is very close to the loop. Our analysis of the 5S and 23S rRNA datasets also revealed neighbor effects using the same parameter setting (complete list in Table S5).

This observation reveals that nucleotides that do not form a base pair can influence the evolution of other nucleotides that are physically close with one another. While the complete structural and functional significance of these neighbor effects remains to be determined, several studies have revealed that: 1) nucleotides associated with base triples and in proximity to these base triples in and near the D stem in tRNA and group I introns have moderately high covariation values [26], [33] (see Figure S4), 2) experimental studies of the ribosome reveal that the D stem in tRNA is dynamic during protein synthesis [62], [63].

Two other research groups have determined covariations by modeling phylogenetic relationships in bacterial 16S rRNA [37], [38]. A comparison of our results with their new covariations revealed that: 1) A few new pairings were identified with both methods; 2) Some of the nucleotides with a covariation identified with their methods are separated by a minimal distance (ie. neighbor effect) while other nucleotides are separated by a much larger distance in the high-resolution crystal structure. A detailed assessment of the similarities and differences are presented in Table S6.

## Discussion

### Utilizing the Evolution of the RNA Structure to Improve the Covariation Methods

Our previous work, presented many years ago revealed that the sensitivity and accuracy of the covariation analysis can be enhanced with the evolutionary history of the RNA [32]. Our analysis of tetraloops in 16S rRNA revealed that this hairpin with four nucleotides that caps a helix can evolve from one common form of the tetraloop to another many times during the evolution of the RNA [36]. This temporal dimension of the RNA structure can distinguish divergent and convergent evolution of specific regions of the RNA. For these studies, the number of times these positions changed during their evolution was determined after the base pairs and tetraloop were identified. While our preference is to utilize the evolutionary history of the positions in the RNA to identify these base pairs and other structural elements, monitoring these temporal changes is a significant computational challenge.

Two groups have modeled the evolution of each position in RNA to identify positional covariation with some success [37], [38]. The Gutell lab’s new RNA Comparative Analysis Database [40], [46] cross-indexes data from each of the dimensions onto the other dimensions. This creates the opportunity to perform several types of novel analysis, including the phylogenetic event counting used for the covariation analysis presented in this manuscript.

### Implementing a Phylogenetic Event Counting Method, and it’s Overall Comparison with Mutual Information

Analysis presented here reveals that overall our Phylogenetic Event Counting method (PEC) is superior than other methods in the identification of base pairs (Figure 3).PEC/JN-Best is more sensitive and accurate than the mutual information based methods that do not utilize the evolution of the RNA in its calculation (see Figures 4), though it does not identify all pairs identified by mutual information based methods (see Figure 5). The modified MIxy method – MIp, when integrated with the JN-Best method, improves the initial mutual information method. The ratio of the number of base pairs that are uniquely identified with PEC/JN-Best and MI/JN-Bes is 62.5% in the 16S rRNA data set (Figure 5) and 76.0% for the three rRNAs (Table S3). Thus the combination of these two covariation methods results in a significant increase in the number of base pairs found. It also demonstrates that the Joint N-Best also improves the sensitivity and accuracy. Of the base pairs identified with covariation analysis, the vast majority occur in secondary structure helices. A few of the base pairs identified with covariation analysis are in the tertiary structure, this includes non-canonical base pairs, psueudoknots, and base pairs that begin to fold the secondary structure into a three-dimensional structure [30].

### Prediction of Base Pairs with Empirical Rules for RNA Secondary Structure – Helix Extend

An assessment of the conservation diagrams of the three primary forms of life – Bacteria, Archaea, and Eukaryotes [http://www.rna.ccbb.utexas.edu/SAE/2B/ConsStruc/]] reveals a significant amount of sequence conservation within each major phylogenetic domain. Many positions in the bacterial 5S, 16S and 23S rRNA sequences analyzed in this paper that are base paired in the comparative structure model have no variation and no covariation, thus the rationale for the helix extension method (see Figure 6 and 7, Table S4). However, as the number of sequences, and the diversity among those sequences increased, we have determined that nearly every base pair does have a covariation for datasets that include the Bacteria, Archaea, Eukaryotic nuclear encoded and the two Eukaryotic organelles. For the later studies, different sets of alignments – (1) Bacteria, (2) Archaea, (3) Eukaryotes, (4) Bacteria, Archaea, Eukaryotic nuclear encoded, (5) nuclear encoded Bacteria, Archaea, Eukaryotes plus their two organelles – Mitochondria and Chloroplasts – were analyzed to identify covariation for nearly every base pair in the 16S and 23S rRNA structure model [6], [43] [http://www.rna.ccbb.utexas.edu/SAE/2A/nt_Frequency/BP/16S_Model].

### The Purity of the Covariation between the Two Positions that Form a Base Pair, and the Identification Neighbor Effects - Weaker Covariations between Positions that do not form a Base Pair

The purity of these covariations that underlies the prediction of a base pair range from an absolute 1∶1 relationship (ie. only base pairs with a strict covariation are found at a specific location in the structure, e.g. 60% G:C and 40% A:U) to base pairs with an increase in the number and types of exceptions (e.g. 50% G:C, 30% A:U, 10% G:U, 5% A:C, 3% A:A and 2% G:G). While our confidence in the prediction of a base pair is higher when the covariation is very pure, the prediction of a base pair becomes increasingly more difficult as the purity of the covariation decreases (see Figure 8). While the pairs of positions with the strongest covariation scores are nearly always base paired in the RNAs higher-order structure, many base pairs have a lower covariation score. Some of the pairs of positions with similar covariation scores do not form a base pair. Instead due to their close proximity in the high-resolution three-dimensional structure, they form a neighbor effect [26], [33] (see Figure 9 and Table S5). While a complete understanding of these neighbor effects are not known, it has been observed that some neighbor effects in tRNA and group I introns are involved in base triple interactions [26], [33] and could be involved in the fine tuning of tRNA structure in protein synthesis [62].

### The Majority of the Nucleotides that Form Base Pairs in the Tertiary Structure do not Covary with One Another

The prediction of an RNA structure with comparative analysis has one primary underlying assumption – the sequences within the same RNA family will fold into the same general secondary and three-dimensional structure. However, while base pairs are predicted when both positions in an alignment have the same pattern of variation, it was implicitly assumed that the sets of nucleotides that form each of the base pairs in an RNAs secondary and tertiary structure will have similar patterns of variation. Our previous analysis of the high-resolution three-dimensional structure revealed in detail at the Gutell lab’s Comparative RNA Web (CRW) Site [http://www.rna.ccbb.utexas.edu/SAE/2A/nt_Frequency/BP/] and substantiated more recently [62] that the vast majority of the sets of nucleotides that form tertiary structure base pairs do not have similar patterns of variation. Thus while we want to identify all of the base pairs in an RNAs higher-order structure with comparative analysis, the current form of covariation analysis will not identify a high percentage of the tertiary structure base pairs for several reasons: 1) While the different base pair types that covary with one another can form similar conformations when two positions in an alignment have similar patterns of variation (e.g. G:C <-> A:U <-> U:A <-> C:G; C:C <-> U:U; A:G <-> G:A; etc.), base pair types that do not covary with one another (e.g. G:A <-> A:A) can also form a similar conformation [33], [64], [65], [66]. Usually the conformations of the base pair types that do not covary with one another are unable to form within a secondary structure helix due to their non-helical backbone conformation. In contrast the local structure flanking most of the tertiary structure base pairs can accommodate the non-helical backbone conformation associated with base pair types that do not covary with one another; 2) Analysis of the high resolution crystal structure of different tRNAs revealed that similar three-dimensional structures of the tRNA form with different sets of tertiary structure interactions [33]. Thus while sets of analogous positions in the RNAs in the same family usually form base pairs in a secondary structure helix, sets of analogous positions do not always form a tertiary structure interaction; 3) Analysis of the high-resolution crystal structures for the rRNAs during different stages of protein synthesis reveals that the secondary structure remains the same to a first approximation. However the ribosome, and for this discussion, the rRNA, is dynamic. The movement is associated with changes in the tertiary structure interaction [67].

In conclusion, we have developed a more sophisticated phylogenetic event counting based method, utilizing the Gutell lab’s new rCAD system. This method in combination with the enhanced mutual information and helix extension methods creates a suite of programs that are superior to existing programs. It has greater sensitivity and accuracy for the identification of the maximum number of secondary and other higher-order base pairs, and the identification of neighbor effects.

## Methods

### Phylogenetic Events Counting (PEC) Method and rCAD System

Given a set of sequences that are properly aligned to form a high quality multiple sequence alignment (MSA) and phylogenetic relationships between all of the sequences in the MSA, the phylogenetic event counting (PEC) method gauges the evolution of RNA to determine two or more positions in a sequence alignment that have similar patterns of variation. A base pair in the RNAs secondary structure is usually associated with the two positions with a strong covariation. The pseudo code of the PEC algorithm is in Figure S1. The phylogenetic relationships are obtained from the taxonomy tree at NCBI (ftp://ftp.ncbi.nih.gov/pub/tax-onomy/).

The nucleotides of each pair of positions are mapped onto the phylogenetic tree according to the phylogenetic information. A tree-traversal from leaf nodes to root counts all types of changes throughout evolution. The NCBI tree is not a binary tree since each node may have more than two child nodes, therefore a standard variation of Fitch’s maximum parsimony approach adapted for non-binary tree is used to determine the nucleotides of ancestor nodes. The nucleotides of ancestor nodes (equality set) are determined as the type of pair occurring most frequently in all sequences within the sub-tree rooted at that node. Each type of child nucleotide pair, which is different from the equality set, will be counted as a positive event (both positions changed) or a negative event (only one position changed) according to the definition. To avoid bias caused by repeat sampling, each type of child nucleotide pair will only be counted once regardless of its number of occurrence. As a consequence, we only consider the minimum number of variations. The Covariation Percentage of Events (CPE) is calculated as the sum of positive events divided by the sum of positive and negative events. High CPE score indicates strong covariation between the two positions.

The PEC method is implemented on the Gutell lab’s RNA Comparative Analysis Database (rCAD) system. This system stores and cross-indexes multiple dimensions of information including raw RNA sequence, multiple sequence alignment, structure and phylogenetic information [40], [46]. This system supports SQL statements accessing individual rows, columns and cells in an alignment as well as RNA structure and taxonomy information. It provides the basis for novel analysis of the fundamental structural characterizations of RNA, such as covariation analysis and structural statistics at the Gutell lab’s Comparative RNA Web (CRW) Site (http://www.rna.ccbb.utexas.edu/SAE/2D/index.php).

### Other Covariation Measures

Standard *Mutual information* (MIxy) is utilized here as a measure for the coordinated or compensatory mutations between two positions. It has been used in several previous studies of RNA [24], [26], [38], [68]. The MIxy between column x and column y in the alignment is calculated as
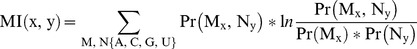
(1)where Pr(M_x_,N_y_) is the joint probability function of nucleotide M and N from column x and y, and Pr(M_x_) and Pr(N_y_) is the marginal probability for a nucleotide (M or N) in column x and y.

An advanced mutual information based method (MIp) was presented by Dunn et al in 2008 [47]. This method estimates the background for each pair of positions, and uses it to remove the influence of entropy and phylogeny. The resulting corrected mutual information (MIp) improves the base-pair identification. We repeated the MIp calculation process described in their research.

An independent method OMES measures the difference between the expected and observed nucleotides frequency for a pair of columns [48]. It is implemented using the formula:
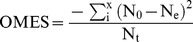
(2)Where N_0_ is the observed number of di-nucleotides in a pair of positions, N_e_ is the expected number, N is the total number of possible di-nucleotide pairs, and N_t_ is the total number of sequences in the alignment.

The calculation of McBASC [50] and ELSC [49] is implemented using the code provided by the authors (http://www.afodor.net/).

### The Joint N-Best Strategy

In 1992, a simple ranking of the highest to lowest mutual information values for tRNA revealed that the top 19 pairings are base pairs in the tRNA secondary structure [26]. And the 20th pairing was a tertiary base pair. However many pairings that are not base paired in the tRNA higher-order structure have higher mutual information values than several of the base pairs in the tRNA secondary structure model. It was determined that the mutual information score has an association with Shannon’s information entropy [69]; The mutual information between two positions is the difference between the sum of the entropies of those two positions minus the joint entropy [http://sciencehouse.wordpress.com/2009/08/08/information-theory/]. At one extreme, a position that only has one type of nucleotide has the minimum entropy (i.e. – highly conserved), while positions that are highly variable (e.g. equal percentages of the four nucleotides) have the maximum entropy. Thus the mutual information value for two positions that have identical patterns of variation (i.e. Covariation) is greater when the entropy value is smaller (ie. greater variation). Thus a simple, although not the most mathematically eloquent means to correct for this, is to determine the positions with the highest mutual information scores, or covariation for each individual position. This simple method, named N-Best was utilized in 1992 to enhance the interpretation of base pairs from the mutual information scores [26].

A variation of N-Best method, Joint N-Best, is used to determine the pairs of nucleotides with the best covariation scores. For each pair (X_1_:Y_1_), the N-Best score of position X_1_ is measured as the ratio of the second highest covariation scores (MIxy, MIp, CPE etc) divided by the highest covariation score in the series of pairs (X_1_:Y_1,_ X_1_:Y_2, ……,_ X_1_:Y_n_ ). The N-Best score of position Y_1_ is measured in the same manner from the series of pairs (X_1_:Y_1,_ X_2_:Y_1, ……,_ X_n_:Y_1_ ). The pairs with both N-Best scores lower than the predefined threshold (≤0.5) will be considered as candidate base-pairs having significant covariations.

### Helix-extension Strategy

Our ultimate goal is to identify every base pair in the RNAs higher-order structure with covariation analysis. However, depending on the RNA dataset, some of the nucleotides that form a base pair might be invariant, or nearly so, and thus they cannot be identified with covariation analysis. Our helix-extension strategy, first implemented in 1980 [20] simply increases the length of a putative helix with G:C, A:U, and G:U base pairs that are 1) adjacent and antiparallel with base pairs with a significant covariation (also referred to as a “nucleation pair”) and 2) occur in at least 85% of the sequences.

### Calculation of Conservation Score and Purity Score

The conservation score for each column of the sequence alignment is calculated with a variation of the Shannon equation,
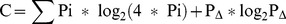
(3)where *Pi* is the frequency of occurrence of base i at a given column and *P_Δ_* is the frequency of deletions (gaps) at that column [6].

The *purity score* measures the extent that each nucleotide – A, U, C, and G, is only associated with one other nucleotide. For example, the following sets of paired nucleotides have the highest purity score –100%: {A:A; G:G; C:C; and U:U}; {A:U; G:C; U:A; C:G}; {A:U; G:G}; {A:A; G:G}; {A:G; G:A}; {A:C; G:U}. The set {A:U; G:C; G:U; and C:G} would have a lower purity score since G is associated with C and U, and the set {A:A, A:G; A:C; and A:U} would have the lowest purity score since A is associated with four different nucleotides. The first group of examples with 100% purity has several sets of perfect covariations, the second lower purity score has a few covariations with one anomaly, while the last set with the lowest purity score has no covariations. Using the procedure described in the Figure S7 that defines the list of base pair types that have a covariation with one another the purity score is the sum of the ranked percentages of base-pairs that have a covariation with the other base pairs. An example showing each step in the calculation of the purity score for two columns in the sequence alignment is presented in Figure S7. When only the overall frequencies of the base pairs are considered, a G:U base pair is not a covariation when the same two positions have a higher percentage of A:U and G:C base pairs. However, since G:U base pairs, also called the wobble base pair [70] occur within a regular helix, a GU-Plus purity score is calculated with a slightly modified procedure; The base pairs G:U (or U:G) are counted as a covariation with G:C (or C:G) and A:U (or U:A) for all of the known base pairs in Figure 8 right side.

### Identification of “Neighbor Effects” and Physical Distance Calculation

The “neighbor effects” are identified with the standard one-directional N-Best method with some constraints. Our objective here is to identify pairs that have not already been identified as a potential base pair, but those pairs that still have a significant covariation. Those pairing are not necessarily indicative of a base pair, instead they comprise a constraint on the evolution of a set of nucleotides. For a pair (X_1_, Y_1_), the N-Best score is calculated as the ratio of the two highest CPE scores in the series of pairs (X_1_:Y_1,_ X_1_:Y_2, ……,_ X_1_:Y_n_ ). This analysis is composed of three steps. The pairs with: 1) N-Best scores exceeding the predefined threshold (0.85); 2) Covariation score (CPE) higher than a predefined lowest cutoff (25%); 3) Total events (positive plus negative) higher than a minimum event threshold, will be considered as neighbor effects.

When the covariation score (CPE) is low (for example pairs with CPE <20%), the background noise could interfere with the signal severely. To remove this noise, only those pairs with a CPE score higher than a predefined lowest cutoff value (25%) will be considered as neighbor effects. The total events threshold is the minimum number of changes at the pair of positions during the evolution of the RNA.

For all neighbor effects, the atomic level physical distance between the atoms are estimated from the three-dimensional high-resolution crystal structure (16S rRNA:,PDBID 1J5E; 5S and 23S rRNA: PDBID 2AW4). The physical distance between two nucleotides is calculated as the Euclidean distance between the centers of atoms that usually form the hydrogen bonds in two nucleotides that are base paired (equation 4).
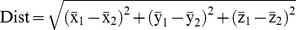
(4)


### Dataset and Filtration Process

The Multiple Sequence Alignments (MSA) of bacterial 16S rRNA (Dataset S1), 5S rRNA (Dataset S2) and 23S rRNA (Dataset S3) are used in this analysis. Considering a MSA consisting of m columns and n rows, the total number of pairwise comparison is N*(N-1)/2 and the time complexity of the PEC algorithm is in the order of O(m^2^n).

Positions that have similar conservation values (Shannon’s information entropy) have the potential to have a higher mutual information score (Figure S5). Thus, the number of pairwise comparison calculations can be reduced significantly by analyzing only those sets of positions with similar conservation values. A coarse filter based on relative conservation score and the mutual information measurement is applied to eliminate the comparisons between two columns in the alignment that will have an insignificant covariation score, which will significantly reduce the computational time for this step of the analysis. The PEC analysis was only performed on those pairwise sets of nucleotides with: 1) the relative entropy score lower than a predefined threshold (0.2) and 2) mutual information value between column X and column Y are among the top 100 of (Column X : any other column) and (any other column : Column Y) [46]. For 16S rRNA, this coarse filter helps to reduce the total of approximately 5,234,230 pairwise comparisons to 14,276 pairings. This smaller number of pairings is analyzed with the PEC method (A complete list in Table S2). The same filtration procedure is applied in processing the 5S rRNA and 23S rRNA data sets.

Among the 608 base pairs (both secondary and tertiary base pairs) in the *T. thermophilus* 16S rRNA high resolution crystal structure, 218 are eliminated in the coarse filter. Almost all of these eliminated base pairs have weak covariations, while only 1 of them can be identified with the PEC/JN-Best. Thus the coarse filter effectively decreases the computational cost by a factor of over 300 with a minor decrease in sensitivity.

### Supplemental Material

All supplemental figures and tables are also available online at: http://www.rna.ccbb.ute-xas.edu/SIM/4A/Phylogenetic_Event_Counting/.

## Supporting Information

Figure S1 **Pseudo code of phylogenetic event counting algorithm.**
(EPS)

Figure S2 **Variation/covariation analysis of the secondary structure of the bacterial 16S rRNA sequence alignment.** Total variation in each pairwise set of sequences (X-direction) is plotted vs. (1) the amount of variation in that set of sequences for the two positions that are base paired in the secondary structure (blue), (2) only one position of the two that are base paired in the secondary structure (red), and (3) variation in the unpaired region of the second structure (green) (Y-direction). The slope, Y-intercept, and R^2^ co-efficiency values of the linear regression line for each of the three analyses are at the right side of the line.
(EPS)

Figure S3 **Graphical representation of N-Best method.** While the mutual-information (MIxy) covariation method compares all positions against all other positions, the N-best method ranks covariation scores for two positions for each individual position. The position numbers are in the X-axis and the MIxy values are in the Y-axis. (A) Left: The MIxy scores for position 3 with all 76 positions in tRNA; Right: The MIxy values for position 13 with all 76 positions are also displayed in the right side with the same manner. (B) Each nucleotide position in a tRNA is shown in the X-axis while the MIxy score are displayed in the Y-axis. The vertical bar is the MIxy value for position Z and each of the individual positions in the X-axis. When the positions with the best covariation scores for each position are base paired in the tRNA structure, that vertical bar is shown in red. The positions with lower MIxy values are shown as black vertical lines. This diagram illustrates that the majority of all positions that are base paired has a MIxy value significantly higher than the MIxy value for all of the other positions.
(EPS)

Figure S4 **The secondary (A) and three-dimensional structure (B) of** ***S. cerevisiae Phe*** **tRNA with neighbor effect identified in 1992.**
(EPS)

Figure S5 **The underlying principle of coarse filter that reduce the number of pairwise comparison.** (**A)** The conservation scores for all nucleotides that are base paired in the 16S rRNA comparative structure model. Each base pair is represented with a colored circle, where the color indicates the purity score (minimal value: 0.472; maximum value: 1). The vast majority of the dots representing base pairs are close to the diagonal. (**B)** The conservation scores for each nucleotide position from 138 to 205 which is under the shadow on the entire *Escherichia coli* 16S rRNA secondary structure (right). The red and blue lines indicate the outer and inner boundaries of the helices respectively while grey lines connect the positions that form a base pair.
(EPS)

Figure S6 **Base pairs in the Bacterial 16S rRNA structure model that are identified with the helix extension method using different nucleation pairs.** Red: true positive base-pairs identified in Joint N-Best method, which are used as nucleation points in the helix extension Magenta: false positives in the nucleation pairs; Blue: true positive base-pairs identified with the helix-extension method; Yellow: false-positive pairs identified with the helix-extension method. Secondary base-pairs are represented by closed circles while tertiary base-pairs are represented by open circle and highlighted with arrows. (A) Using pairs identified in PEC/JN-Best as the nucleation pairs. (B) Using pairs identified in MI/JN-Best as the nucleation pairs.
(EPS)

Figure S7 **Example of the determination of a purity score.** For a given pair of positions in the alignment, all base-pair types are ranked by their frequency, from the highest to the lowest as shown in the middle. Starting from the highest ranked base-pair type, each base-pair type is processed to determine if both positions change (ie. covariation). The sum of the percentages of the base pair types that are a covariation (red circles) are calculated as the purity score for this set of positions. The base pairing frequency matrix is rearranged during this process. The most frequent nucleotides are first placed as the top 3′ nucleotide and leftmost 5′ nucleotide. Subsequently the 5′ and 3′ nucleotides that form a covariation pair are placed in descending order, resulting in the placement of the base pairs that covary along a diagonal.
(EPS)

Figure S8 **The maximal distance between the positions defined to be a neighbor effect is determined from a comparison of the number of phylogenetic events.** Different phylogenetic events and their number of positions with different physical distances were calculated. Those positions with at least 10 phylogenetic events contain a large number of positions that are very close in three-dimensional space and a very small number of positions with larger physical distances.
(EPS)

Table S1 **The phylogenetic distribution and sequence similarities of the 16S, 5S and 23S rRNA datasets used in analysis.**
(XLS)

Table S2 **Detail information about all 14276 pairs of columns process in Phylogenetic Event Counting analysis on 16S rRNA data set.**
(XLS)

Table S3 **The unique and common pairs identified by PEC/JN-Best, MIxy/JN-Best and MIp/JN-Best using the 16S, 5S and 23S rRNA data sets.**
(XLS)

Table S4 **The complete list of nucleation pairs and extended pairs involved in the Helix Extension analysis on the 16S, 5S and 23S rRNA data sets.**
(XLS)

Table S5 **A complete list of neighbor effects identified with our analysis.**
(XLS)

Table S6 **The evaluation of the “new putative interactions in 16S Rrna” discovered by 2 other groups.**
(XLS)

Dataset S1 **The bacterial 16S rRNA sequence alignment.**
(FASTA)

Dataset S2 **The bacterial 5S rRNA sequence alignment.**
(FASTA)

Dataset S3 **The bacterial 23S rRNA sequence alignment.**
(FASTA)
